# The impact of long-term trends in continuity of care on the medical expenses of hypertensive patients: based on group-based trajectory model

**DOI:** 10.3389/fpubh.2025.1598324

**Published:** 2025-07-30

**Authors:** Yanqiu Du, Di Liang, Gaofeng Zhang, Yongsong Luo, Jiayan Huang, Yin Dong

**Affiliations:** ^1^School of Public Health, Fudan University, Shanghai, China; ^2^Key Lab of Health Technology Assessment (Fudan University), National Health Commission, Shanghai, China; ^3^Dean's Office, The People's Hospital of Yuhuan, Taizhou, Zhejiang, China; ^4^Medical Administration Department, Health Commission of Yuhuan, Taizhou, Zhejiang, China

**Keywords:** hypertension, continuity of care (COC), medical expenses, group-based trajectory modeling (GBTM), long-term trends

## Abstract

**Purpose:**

This study aimed to identify long-term trends in continuity of care (COC) among hypertensive patients using group-based trajectory modeling (GBTM) and evaluate their association with medical expenses, thereby providing evidence for chronic disease management.

**Methods:**

We analyzed 6-year (2016–2021) reimbursement data of the social health insurance from Yuhuan City, China, including 30,545 hypertensive adults. Continuity of Care Index (COCI) was calculated annually. GBTM was employed to classify patients into trajectory subgroups based on COCI trends, with the best-fitting model selection guided by Bayesian information criterion (BIC), average posterior probability (AvePP). Multiple linear regression assessed the relationship between trajectory groups and annual medical expenses, adjusting for age, gender, insurance type, and Charlson Comorbidity Index (CCI).

**Results:**

Four COCI trajectories were identified: low-level maintenance (52.06%), low-level increase (17.14%), high-level decrease (18.94%), and high-level maintenance (11.87%). Patients in the high-level maintenance group incurred the lowest annual medical expenses (mean range: ¥3,786–¥5,088), while the low-level maintenance group exhibited the highest (mean range: ¥6,450–¥10,321). After adjustment, the low-level maintenance group had significantly higher expenses than the high-level maintenance group (β = 3,049.44 CNY, *p* < 0.001). Older age, employee insurance coverage, and higher CCI were also associated with increased medical expenses (*p* < 0.001).

**Conclusion:**

Sustained high continuity of care correlates with reduced medical expenses in hypertensive patients. Long-term COC maintenance should be prioritized in chronic disease management to mitigate healthcare costs. Policymakers should incentivize care continuity through integrated health systems and targeted patient interventions.

## Introduction

The hypertension burden is continuously increasing globally, especially in the WHO Asia—Pacific and Southeast Asia regions. The percentage of hypertensive adults in the WHO European region decreased in 2019 compared to 1990. In Asian regions, it increased, especially in the WHO Western Pacific Region (from 24 to 28%; including Australia, New Zealand, China, etc.) and the number of hypertensive adults in the WHO Western Pacific region more than doubled from 1990 to 2019 (144 million to 346 million) (1). The medical cost burden arising from the long-term management of these patients has also become one of the challenges facing these countries (2). These statistics emphasize the importance of enhancing awareness and management of hypertension.

The 2018 World Health Organization (WHO) implementation strategy of integrated people-centered health services underscores the significance of continuity of care (3). Patients suffering from chronic diseases, including hypertension and diabetes, have the potential to benefit from the enhanced continuity of care that is characteristic of an integrated health system (4–6). Some countries (such as Australia, China, and New Zealand) are actively promoting the realization of integrated services, thereby enhancing the providers' ability to deliver continuity of care (7, 8). However, from the perspective of the patients, the continuity of care is inconsistent (9). This inconsistency is not only the difference between individual patient's health behavior, but also the change of continuity brought about by the same individual's behavior change over a long period of time.

Currently, most studies on continuity of care measurement have relatively short observation periods, typically ranging from 1 to 3 years (10–12), and are often limited by small sample sizes (9, 13). Shorter observation periods mean fewer data points for each individual, and there is a notable lack of long-term observations on trends in continuity of care at the individual level. Consequently, there is insufficient research categorizing individuals with different trends into distinct patient subgroups. This gap significantly weakens the evidence base for evaluating the effectiveness of service continuity over extended periods, particularly for the long-term management of chronic diseases. It also limits our ability to demonstrate the correlation between continuity of care and healthcare costs using large-scale real-world data. However, when addressing the dual limitations of short observation periods and small sample sizes, a significant challenge arises: identifying appropriate statistical tools to efficiently process long-term, large-sample data.

This study attempts to construct the group-based trajectory models to identify subgroups of hypertensive patients with different trends in continuity over a long period of time, and examines whether there are differences in medical expenses between different subgroups with different continuity trends, thereby informing policies to incentivize long-term care continuity through integrated health systems and patient engagement programs, which may reduce healthcare costs.

## Methods

### Data source

The research data was sourced from the reimbursement data of social health insurance (SHI) in Yuhuan City (2016–2021). Yuhuan City is a coastal city in Zhejiang Province, China. Yuhuan City is representative in terms of integrated care and universal health insurance coverage. the city has a permanent population of ~648,000, and the medical insurance participation rate is 99.86% (14).

The SHI data obtained in this study contains the medical records of all insured people in Yuhuan city. The SHI data encompasses individuals enrolled in the basic medical insurance for urban and rural residents and the basic medical insurance for urban workers. The health institutions include all second- and third-level hospitals, community health service centers, township hospitals, village clinics, pharmacies, and private clinics. As long as individuals participate in the SHI in Yuhuan City, their reimbursement records will be included in Yuhuan City's SHI data, regardless of whether they receive medical treatment at health institutions in Yuhuan City, in other cities of Zhejiang Province, or in other provinces of China. For a detailed description of the original variables included by the reimbursement data, please refer to the supplementary 1.

### Research samples

The inclusion criteria were patients with a diagnosis of hypertension, as identified based on the International Classification of Diseases, Tenth Revision (ICD-10) code in their admission records. The Patients whose main diagnostic codes contained the following ICD-10 codes were identified as hypertensive patients: I10, I11, I12, I13, I15.

The exclusion criteria were patients with fewer than 3 years in which the number of annual visits was greater than or equal to three. This criterion was based on data requirements for the continuity of care index calculation and trajectory model fitting (9, 15, 16). In addition, the individuals under the age of 18 at the starting point of the data (January 1, 2016) were also excluded.

### Data analysis

**Figure 1:**
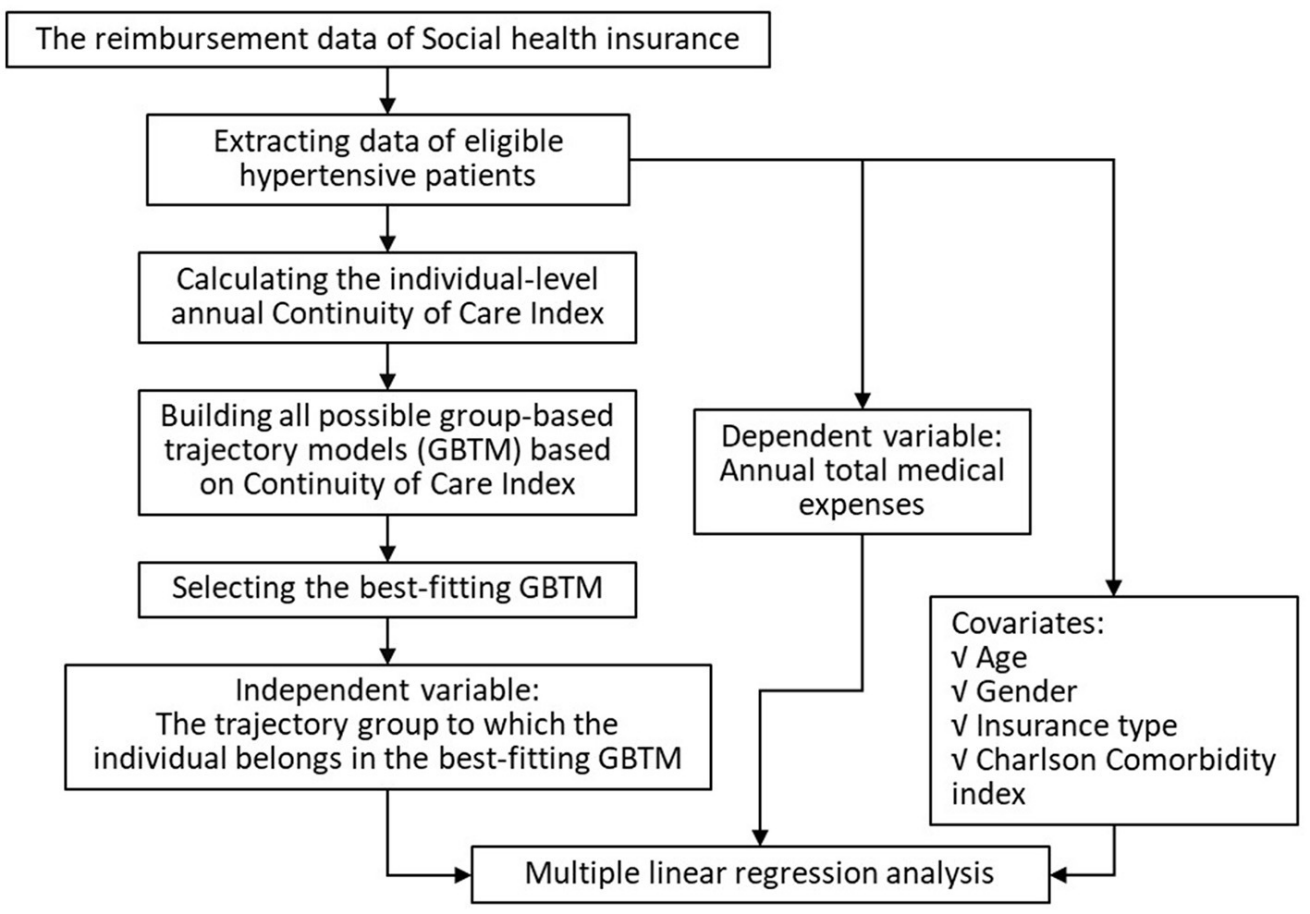
Data analysis process.

The data analysis mainly includes calculating the individual-level Continuity of Care Index (COCI) and Charlson Comorbidity Index (CCI), constructing the group-based trajectory models (GBTM) and selecting the best-fitting model, and performing multiple linear regression analysis. The data analysis process primarily comprised three components: 1) calculation of the COCI at the individual level, 2) construction of group-based trajectory models (GBTM) based on COCI and selection of the best-fitting model, and 3) multiple linear regression analysis to evaluate associations between trajectory groups and medical expenses (Figure 1).

### Continuity of Care Index (COCI)

The COCI was employed to assess individual continuity of care, signifying whether a patient consulted rates with the same health care provider (6). In this study, a health institution was designated as a service provider. The COCI is calculated as follows.


$$\begin{array}{l} COCI=\;\frac{\sum_{i=1}^{p}n_{i}\left(n_{i}-1\right)}{N\left(N-1\right)} \end{array}$$

Where *N* is the total number of visits and *n*_*i*_ is the number of visits to the provider (*i*). Theoretically, the COCI ranges from 0 to 1, with higher values indicating stronger continuity of care.

### Charlson Comorbidity Index (CCI)

It is important to note that hypertensive patients frequently have comorbidities, and this study employs the Charlson Comorbidity Index (CCI) as a covariate. The CCI is a method for calculating an index that represents an individual's total disease burden or mortality risk (17). The index is calculated by classifying the comorbidity status and assigning different weights to each status based on its risk associated with mortality, and then summing the weights. The Charlson Comorbidity Index is widely used in epidemiological studies to adjust (control) comorbidity. An updated Charlson Comorbidity Index (upCCI) was published in 2011 (17). The upCCI recalibrates the weight of each condition by taking into account the treatment prognosis of the original 19 disease conditions under contemporary medical circumstances. The upCCI in this study was computed using the Excel-based calculation tool released by Prommik in 2022 (see supplementary 2) (18). The upCCI encompassed a range of diseases, including myocardial infarction, congestive heart failure, peripheral vascular disease, cerebrovascular disease, dementia, chronic pulmonary disease, among others.

### Group-based trajectory models (GBTM)

To ascertain the most suitable discrete number and patterns of continuity trend, we employed a GBTM analysis, a frequently utilized approach in health research for identifying potential subgroups within a population (15, 16). The time metric corresponded to the number of years since baseline (2016–2021), and the GBTMs were constructed using the COCI of the patient over a 6-year period. The selection criteria for selecting the best-fitting model from all GBTMs in this study were as follows. (1) Bayesian information criterion (BIC), which indicates a best-fitting model when its value is close to 0; (2) The proportion of individuals included in each trajectory group, which must be ≥5%; (3) The average posterior probability (AvePP), which reflects the degree of conformity of the members of the subgroup to the trajectory after grouping, and is generally considered acceptable if it is >0.7; (4) the trajectory shape is of practical significance (19–22).

According to the best-fitting model, patients were assigned to the trajectory group with the highest AvePP.

### Multiple liner regression

Multiple linear regression was used to assess the relationship between the trajectory grouping of individual patients as the independent variable and their annual medical expenses as the dependent variable. The covariates included age, gender, health insurance type, and comorbidity index. Among them, the type of health insurance was used as a covariate to represent their income level. The baseline characteristics of the study population were described in terms of quantitative and percentage for categorical variables. The categorical variables were compared using a chi-square test or Fisher exact test as appropriate. A two-tailed *p* value < 0.05 was considered statistically significant. The analysis was performed using the SAS (version 9.4) software macro “TRAJ” to implement GBTM, and Stata (version 17SE) for other analyses.

Collectively, the application of the COC, GBTM guided by BIC, the updated CCI, and multiple linear regression analyses provided a robust framework for identifying distinct patient subgroups based on healthcare utilization patterns. This integrated approach allowed us to precisely link COC to annual medical expenditures.

### Ethics approval

This study received ethical approval from the Ethics Committee of School of Public Health, Fudan University (IRB#2024-08-1154). All data were anonymized to protect patient privacy.

## Results

### Patient characteristics

Based on 39.87 million reimbursement records over 6 years from the entire city, a total of 30,545 eligible hypertension patients were included in this study. The majority of patients were covered by residents' medical insurance (55.90%, Table 1). The proportion of patients with a comorbidity index of 0 was 52.99%, and the proportion of patients with a comorbidity index greater than or equal to 4 was < 1%. The total annual per capita medical expenses of hypertensive patients showed an overall increasing trend. With the exception of 2020, which demonstrated negative growth (−5.63%) compared to 2019, the remaining years exhibited positive year-on-year growth.

**Table 1:** Descriptions of baseline characteristics for the overall patients with hypertension.

**Characteristics**	**Total (*N* = 30,545)**
**Gender**, ***n*** **(%)**	
Male	15,688 (51.36)
Female	14,857 (48.64)
**Age groups**, ***n*** **(%)**	
19–59 years	8,786 (28.76)
60–74 years	13,899 (45.50)
75 years old and above	7,860 (25.73)
**Types of medical insurance**, ***n*** **(%)**	
Employees' medical insurance	13,469 (44.10)
Residents' medical insurance	17,076 (55.90)
**Update Charlson Comorbidity index**, ***n*** **(%)**	
0	16,188 (52.99)
1	11,366 (37.21)
2	1,672 (5.47)
3	1,104 (3.61)
≥4	215 (0.70)
**Annual per capita total medical expenses, Yuan (growth rate %)**	
In 2016	2,910.89 (-)
In 2017	3,430.88 (17.86)
In 2018	3,799.14 (10.73)
In 2019	4,515.39 (18.85)
In 2020	4,261.27 (−5.63)
In 2021	4,465.40 (4.79)

### Best-fitting group-based trajectory model

We tested models with 2–6 trajectory groups (supplementary 3). The four-group model was selected as optimal due to its balance of statistical fit (BIC closest to zero), practical interpretability, and subgroup proportions >5%. Models with 5–6 groups included subgroups with < 5% membership (e.g., 4.61% in the 5-group model), violating model stability criteria. The four-group model also exhibited high AvePP (>0.7 for all groups), ensuring robust classification (Table 2). The group trajectory model fitting results, derived from the 6-year continuous index of individual hypertensive patients, indicate that among the 81 models with four trajectories, the sequential combination of one quadratic polynomial and three cubic polynomial trajectory forms (2,3,3,3) is the combination with the BIC closest to 0 among all four trajectory models (supplementary 3). The BIC of all GBTMs and the proportion of individuals in each trajectory group can be found in the supplementary 3.

**Table 2:** Comparison of model performance for the models with different shape orders.

**No. of groups**	**Shape per group**	**BIC**	**Sample proportion per group (%)**						**AvePP**					
2	2,3	−35,676.64	67.73	32.27					0.94	0.90				
3	2,3,3	−32,433.48	50.39	36.67	12.94				0.91	0.93	0.68			
4	2,3,3,3 (Best-fitting)	−29,415.80	52.06	17.14	18.94	11.87			0.92	0.80	0.80	0.88		
5	2,3,3,3,3	−28,407.92	49.90	4.61	16.83	13.93	14.73		0.91	0.59	0.82	0.80	0.85	
6	3,3,3,3,3,2	−27,700.30	47.12	16.15	3.27	14.23	7.55	11.67	0.90	0.80	0.64	0.87	0.61	0.83

Shape Number Definition: 2, quadratic; 3, cubic; BIC, the Bayesian information criterion; AvePP, the average posterior probability.

**Figure 2:**
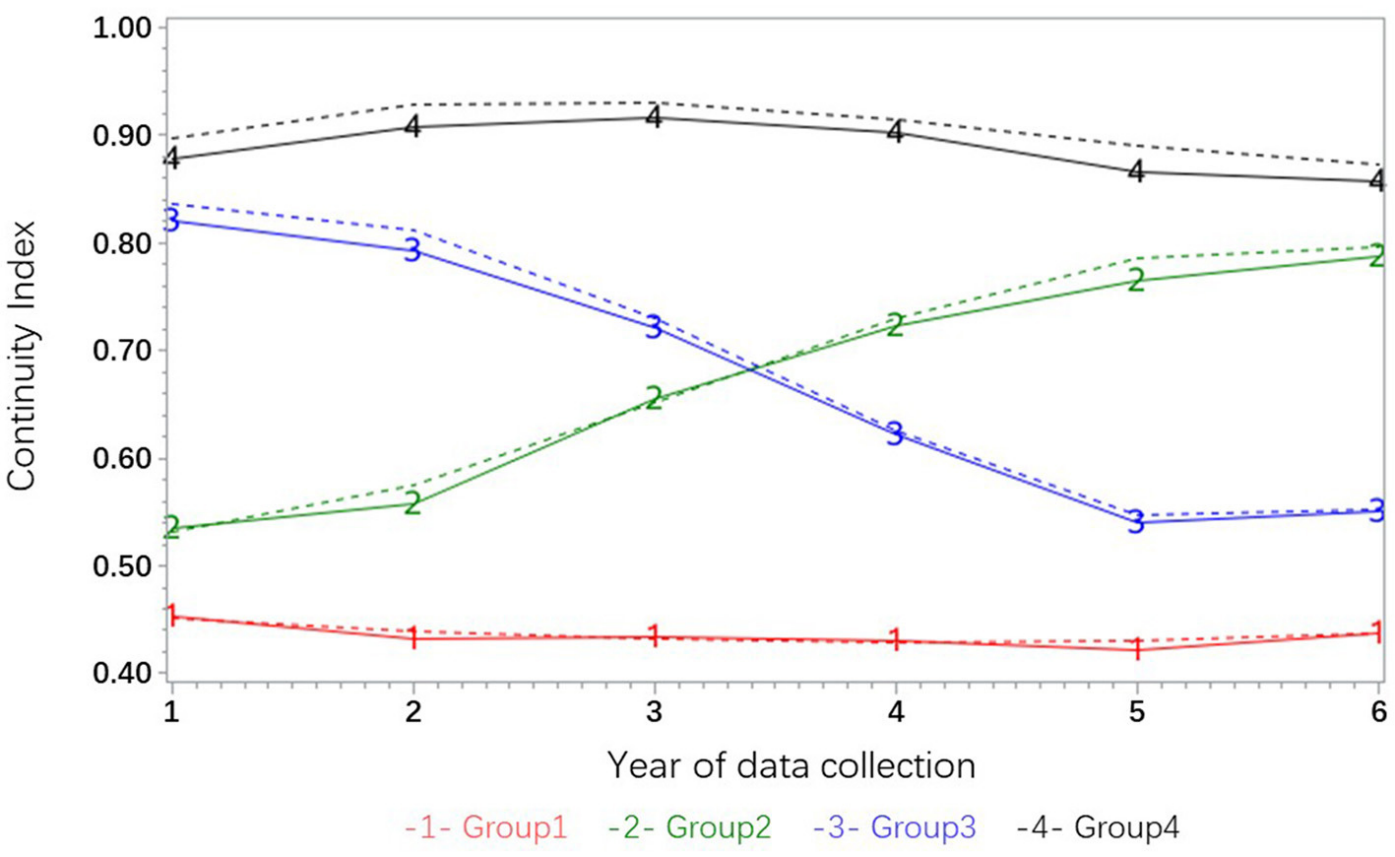
Trajectory subgroups of continuity of care index.

The best-fitting trajectory model for the hypertension group indicates that the COCI of group G1 demonstrates a tendency to persist at a low level, with an index value ranging from 0.4 to 0.5 over the 6-year period (Figure 2). The trajectory of group G2 exhibited an upward trend, rising from ~0.55 to around 0.8, suggesting a sustained rise from a low level. The trajectory of group G3 exhibited a persistent downward trend from a high level, while the trajectory of group G4 fluctuated slightly but remained at a high level.

### Characteristics of the trajectory groups

A comparison of these groups reveals that group G1, characterized by a low level, accounts for the highest proportion of individuals (52.06%), while group G4, which maintains a high level, exhibits the lowest proportion (11.87%, Table 3). An analysis of gender ratio reveals a slight preponderance of males in each trajectory group, with group G1 exhibiting a 4.05% lower proportion of males compared to group G4. The variation in gender ratio among the groups is statistically significant (χ^2^ = 27.20, *p* < 0.001).

**Table 3:** Characteristics among trajectory groups.

**Variables (Item)**	**G1**	**G2**	**G3**	**G4**	***p***
Trajectory pattern	Low-level maintenance	Low-level increase	High-level decrease	High-level maintenance	N/A
*n* (%)	15901 (52.06)	5235 (17.14)	5784 (18.94)	3625 (11.87)	N/A
**Gender**, ***n*** **(%)**					*p* < 0.001
Male	15688 (51.36)	2727 (52.09)	3047 (52.68)	1960 (54.07)	
Female	14857 (48.64)	2508 (47.91)	2737 (47.32)	1665 (45.93)	
**Age groups**, ***n*** **(%)**					*p* < 0.001
19–59 years	8786 (28.76)	1428 (27.28)	1503 (25.99)	871 (24.03)	
60–74 years	13899 (45.50)	2321 (44.34)	2726 (47.13)	1577 (43.50)	
75 years old and above	7860 (25.73)	1486 (28.39)	1555 (26.88)	1177 (32.47)	
**Types of medical insurance**, ***n*** **(%)**					*p* < 0.001
Employees' medical insurance	13469 (44.10)	2000 (38.20)	1999 (34.56)	995 (27.45)	
Residents' medical insurance	17076 (55.90)	3235 (61.80)	3785 (65.44)	2630 (72.55)	
**Update Charlson Comorbidity index**, ***n*** **(%)**					*p* < 0.001
0	16188 (52.99)	3057 (58.39)	3363 (58.14)	2326 (64.16)	
1	11366 (37.21)	1794 (34.26)	1977 (34.18)	1121 (30.92)	
2	1672 (5.47)	238 (4.54)	277 (4.78)	119 (3.28)	
3	1104 (3.61)	123 (2.34)	138 (2.38)	49 (1.35)	
≥4	215 (0.70)	23 (0.43)	29 (0.50)	10 (0.27)	

In terms of age group, the proportion of individuals between the ages of 19 and 59 in trajectory groups G1 to G4 was 31.34, 27.28, 25.99, and 24.03%, respectively, indicating a downward trend. Concurrently, the proportion of individuals aged 75 and above exhibited a downward trend, and the discrepancy in age composition among the trajectory groups was statistically significant (χ^2^ = 223.61, *p* < 0.001).

With respect to health insurance type, the proportion of individuals covered by urban employee medical insurance in group G1 was 53.30%, which was higher than the proportion of individuals covered by employee medical insurance in group G4 (27.45%). A statistically significant difference in the composition of medical insurance was identified between groups G1 and G4 (χ^2^ = 1217.64, *p* < 0.001).

With respect to the comorbidity index, the proportion of individuals with a co-morbidity index of 0 in the G4 high-maintenance group was 64.16%, while the proportion in the G1 low-maintenance group was 46.80%. The difference in the distribution of the co-morbidity index between the G1 and G4 groups was statistically significant (χ^2^ = 676.52, *p* < 0.001). The differences in the distributions of the above characteristic variables among the trajectory groups indicate that the populations in the trajectory groups have different characteristics, and also suggest that these variables should be controlled for in further regression analysis.

Furthermore, a comparison of annual medical expenses among subgroups grouped by trajectory revealed that the G4 high maintenance group exhibited lower annual medical expenses over a 6-year period, while the G1 low maintenance group demonstrated higher expenses compared to the other groups (Table 4).

**Table 4:** Direct comparison of the annual total medical expenses among trajectory groups.

**Annual means (CNY)**	**G1**	**G2**	**G3**	**G4**
In 2016	6,450.26	5,387.74	4,008.61	4,048.39
In 2017	7,554.91	5,922.39	4,351.41	3,786.64
In 2018	8,396.90	5,960.50	5,528.96	4,110.72
In 2019	9,666.27	6,212.73	7,830.79	4,808.25
In 2020	9,320.28	5,275.05	8,135.07	4,228.87
In 2021	10,320.70	5,874.06	8,768.90	5,088.15

CNY, the international standard currency code for the Renminbi (Chinese Yuan).

### The association between trajectory of continuity of care and medical expenses

The findings of the multiple linear regression analysis demonstrate that, after controlling for covariates, among the trajectory subgroups of the independent variables, the coefficient of the G1 low maintenance group, with the G4 high maintenance group serving as the reference, is 3049.44 (Table 5). This indicates that, in comparison with the G4 group, the average annual medical expenses of individuals in the G1 group increased by 3049.44 yuan. This discrepancy is statistically significant (*p* < 0.001). The regression coefficient for the G2 low-level upward group is 989.80 (*p* < 0.001), and the regression coefficient for the G3 high-level downward group is 1800.38 (*p* < This indicates that the average annual medical expenses for the G2 and G3 groups have increased by 989.80 yuan and 1800.38 yuan, respectively, compared to the G4 group. The observed differences are statistically significant.

**Table 5:** The results of multiple linear regression analysis.

**Variables**	**Coefficient**	**Standard error**	***t***	***p***	**95% confidence interval**	
**Trajectory pattern**						
G4 (High-level maintenance)	Ref.	-	-	-	-	-
G1 (Low-level maintenance)	3,049.44	165.45	18.43	< 0.001	2,725.15	3,373.73
G2 (Low-level increase)	989.80	190.56	5.19	< 0.001	616.3	1,363.3
G3 (High-level decrease)	1,800.38	186.71	9.64	< 0.001	1,434.42	2,166.34
**Gender**						
Female	Ref.	-	-	-	-	-
Male	445.52	104.50	4.26	< 0.001	240.69	650.35
**Age groups**						
75 years old and above	Ref.	-	-	-	-	-
19–59 years	−4,261.22	144.57	−29.47	< 0.001	−4,544.59	−3,977.85
60–74 years	−2,237.08	124.87	−17.92	< 0.001	−2,481.83	−1,992.33
**Types of medical insurance**						
Residents' medical insurance	Ref.	-	-	-	-	-
Employees' medical insurance	3,563.46	114.17	31.21	< 0.001	3,339.68	3,787.24
Update Charlson Comorbidity index	2,284.92	61.60	37.09	< 0.001	2,164.18	2,405.65
Intercept	4,144.91	174.52	23.75	< 0.001	3,802.85	4,486.97

Among the covariates, the gender variable employs females as the reference group, and the coefficient for males is 445.52 (*p* < 0.001). The age group variable employs the ≥75 age group as the reference, and the coefficient for the 19–59 age group is −4231.22 (*p* < 0.001); the coefficient for the 60–74 age group is −2237.08 (*p* < 0.001). The analysis further revealed that the coefficient for employee medical insurance was 3563.46 (*p* < 0.001). The coefficient of the comorbidity index variable (continuous variable) was 2284.92 (*p* < 0.001).

## Discussion

In this study, we utilized 6 years of medical insurance reimbursement data (2016–2021) from a city. We employed a group-based trajectory model (GBTM) to analyze the service continuity trajectories of a hypertensive patient group, identifying four subgroups: low maintenance, low increase, high decrease, and high maintenance. This study indicated that, among all subgroups, the patient subgroup with consistently high CCI had the lowest annual medical expenses.

This finding aligns with the conclusions of domestic and foreign studies by Harshita Kajaria-Montag (2024), Shang Chunxiao (2023), Di Liang (2022), and Anna Nicolet (2022) (9, 13, 23, 24). Moreover, based on the GBTM, this the study indicated that individuals whose continuity increased from a low level of continuity, exhibited higher medical expenditures compared to the group that had sustained a high level of continuity. The underlying reason for this result may be that healthcare-seeking behavior (continuity of care), while capable of rapid change, might require a longer time frame to demonstrate the effects of such changes. Therefore, this finding highlighted the importance of maintaining a high level of continuity over the long term in managing chronic diseases to control medical costs effectively.

The comparison of characteristics between trajectory subgroups reveals that groups with divergent continuity trends exhibit disparities in gender, age group, type of medical insurance, and other demographics. These distinctions in individual characteristics not only facilitate the depiction of group characteristics for trajectory subgroups and the analysis of their medical preferences but also offer a multitude of entry points and more precise population orientation for the formulation and implementation of strategies to enhance continuity. Future research endeavors should explore and examine additional patient characteristics to ascertain which individual characteristics of patients are more likely to maintain a high level of continuity of services over an extended period.

### Limitations

The study is subject to several limitations. Firstly, while COCI measures provider consistency, it does not capture care quality or appropriateness. Therefore, this study does not emphasize the association between a high level of continuity and service quality. Instead, it focuses on the correlation between the change trajectory of COCI and the actual medical expenses incurred by patients. Secondly, medical reimbursement data is only capable of reflecting service continuity at the institutional level, precluding the analysis of continuity at the individual physician level, potentially overestimating continuity.

## Conclusion

Sustained high continuity of care over 6 years is associated with 30%−50% lower medical expenses in hypertensive patients, independent of age, comorbidities, and insurance type. Policymakers should prioritize financial incentives for providers and patients to maintain long-term care relationships, particularly in low-continuity subgroups.

## Data Availability

The raw data supporting the conclusions of this article will be made available by the authors, without undue reservation.
