# Supplementary material for: The impact of long-term trends in continuity of care on the medical expenses of hypertensive patients: based on group-based trajectory model
Source: Front Public Health. 2025 Jul 30;13:1598324. doi: 10.3389/fpubh.2025.1598324 (PMC12343502; doi:10.3389/fpubh.2025.1598324)
Supplement: Supplementary file 1 [file Table_1.DOCX]

**Original variables in the social health insurance reimbursement data obtained in this study:**

1. Personal code (unique identity code)

2. Gender

3. Date of birth

4. Name of insurance type

5. Number of health institution

6. Name of health institution

7. Visit date

8. Discharge date (for inpatient)

9. Type of visit (outpatient, inpatient, emergency)

10. Disease upon admission (disease name)

11. Disease code upon admission (International Classification of Diseases, ICD-10)

12. Disease upon discharge (name)

13. Disease code upon discharge (ICD-10)

14. Total medical expense (The value of the total medical expense is the sum of the total amount within the medical insurance scope and the out-of-pocket amount)

15. Total amount within the medical insurance scope (The total amount within the medical insurance scope is the sum of the medical insurance reimbursement amount and the amount borne by the individual for medical insurance)

16. Out-of-pocket amount (Payment amount for out-of-insurance items)

17. Medical insurance reimbursement amount

18. Amount borne by the individual for medical insurance (The remaining individual-borne part after the reimbursement of medical insurance items)
